# An empirical study of fault localisation techniques for deep neural networks

**DOI:** 10.1007/s10664-025-10657-7

**Published:** 2025-06-10

**Authors:** Nargiz Humbatova, Jinhan Kim, Gunel Jahangirova, Shin Yoo, Paolo Tonella

**Affiliations:** 1 Università della Svizzera italiana, Via Buffi, 13, Lugano, Switzerland; 2 King’s College London, Bush House, Strand Campus, 30 Aldwych, London, UK; 3 KAIST, 291 Daehak-ro, Yuseong-gu, Daejeon, Republic of Korea

**Keywords:** Deep learning, Real faults, Fault localisation

## Abstract

With the increased popularity of Deep Neural Networks (DNNs), increases also the need for tools to assist developers in the DNN implementation, testing and debugging process. Several approaches have been proposed that automatically analyse and localise potential faults in DNNs under test. In this work, we evaluate and compare existing state-of-the-art fault localisation techniques, which operate based on both dynamic and static analysis of the DNN. The evaluation is performed on a benchmark consisting of both real faults obtained from bug reporting platforms and faulty models produced by a mutation tool. Our findings indicate that the usage of a single, specific ground truth (e.g. the human-defined one) for the evaluation of DNN fault localisation tools results in pretty low performance (maximum average recall of 0.33 and precision of 0.21). However, such figures increase when considering alternative, equivalent patches that exist for a given faulty DNN. The results indicate that DeepFD is the most effective tool, achieving an average recall of 0.55 and a precision of 0.37 on our benchmark.

## Introduction

Fault localisation (FL) for DNNs is a rapidly evolving area of DL testing (Wardat et al. 2021, 2022; Cao et al. 2022; Nikanjam et al. 2021; Schoop et al. 2021). The decision logic of traditional software systems is encoded in their source code. Correspondingly, fault localisation for such systems consists of identifying the parts of code that are most likely responsible for the encountered misbehaviour. Unlike traditional software systems, however, the decision logic of DL systems depends on many components such as the model structure, selected hyper-parameters, training dataset, and the framework used to perform the training process. Moreover, DL systems are stochastic in nature, as a retraining with the exactly same parameters might lead to a slightly different final model and performance. These distinctive characteristics make the mapping of a misbehaviour (e.g., poor classification accuracy) to a specific fault type a highly challenging task.

Existing state-of-the-art works (Wardat et al. 2021; Zhang et al. 2021; Wardat et al. 2022; Baker et al. 2022; Nikanjam et al. 2021) that focus on the problem of fault localisation for DL systems were shown to be adequate for this task when evaluated on different sets of real-world problems extracted from StackOverflow and GitHub platforms or were deemed useful by developers in the process of fault localisation and fixing (Schoop et al. 2021). However, these approaches rely on patterns of inefficient model structure design, as well as a set of predefined rules about the values of internal indicators measured during the DL training process. This makes the effectiveness of these approaches highly dependent on the identified set of rules and on the threshold values selected to discriminate the values of the internal indicators of a fault.

To understand whether these tools effectively generalise to a diverse set of fault types and DL systems, and thus, are effective for the real-world usage, we performed an empirical evaluation on a curated benchmark of carefully selected subjects. In this benchmark, the faults obtained by the artificial injection of defects into otherwise well-performing DL models are combined with a set of reproduced real-world DL faults. We ensured that our evaluation involves models of different structure and complexity that solve problems from different domains. The existing evaluations of FL tools are performed only on datasets in which there is a single ground truth repair for each fault. However, an improvement in the performance of a DL model can be achieved by applying different but equally effective fixes. Therefore, limiting the evaluation to a single ground truth might lead to correctly suggested alternative fixes being classified as incorrect, which posits a significant threat to the validity of the performed experiments. To address this issue, we perform a *neutrality analysis*, which aims to identify multiple alternative patches that fix a fault in the DL model. We then evaluate the FL tools considering not only the manually identified single ground truth but also all the available fixes.

Our results show that existing DNN FL techniques produce stable results in a relatively short time, ranging from an average of 7 to 278 seconds. However, the accuracy of fault localisation techniques with regard to ground truth provided for each issue is quite low (with a maximum average recall of 0.33 and a precision of 0.21). Once we extend the available ground truth to the changes that produce equivalent or superior improvement in the model’s performance, the fault localisation performance significantly improves (the observed maximum recall growth is from 0.33 to 0.55). This shows that the evaluation of the approaches on only one variant of the ground truth does not indeed provide accurate results.

The results indicate that the highest FL performance is achieved by DeepFD, which is also the tool that requires the longest execution time. Neuralint is extremely efficient, as it is based on static analysis and does not require model training, although its performance is inferior to that of DeepFD.

In general, we make the following contributions in this paper:

- An empirical evaluation of four state-of-the-art fault localisation tools on a set of real-world and artificially injected faults, in terms of fault detection effectiveness, efficiency and stability of the results (both considering a single, fixed ground truth, as well as multiple ground truths obtained by means of neutrality analysis).
- The largest and augmented dataset of reproducible DNN faults with an extended ground truth based on neutrality analysis.
- An analysis of the output messages of fault localisation tools in terms of actionability.

## Background

Most of the proposed approaches for fault localisation for DL systems focus on analysing the run-time behaviour during model training. According to the collected information and some predefined rules, these approaches decide whether they can spot any abnormalities and report them (Wardat et al. 2021, 2022; Schoop et al. 2021).

### DeepLocalize and DeepDiagnosis

During the training of a DNN, DeepLocalize (DL) (Wardat et al. 2021) collects various performance indicators such as loss values, performance metrics, weights, gradients, and neuron activation values. The main idea behind this approach is that the historic trends in the performance evaluation or the values propagated between layers can serve as an indicator of a fault’s presence. To allow the collection of the necessary data, a developer should insert a custom callback provided by the tool into the source code regulating the training process. A callback is a mechanism that is invoked at different stages of model training (e.g., at the start or end of an epoch, before or after a single batch Chollet et al. (2015)) to perform a set of desired actions – store historical data reflecting the dynamics of the training process in our case. The tool then compares the analysed values with a list of pre-defined failure symptoms and root causes, which the authors have collected from the existing literature. Based on the performed analysis, DeepLocalize either claims that the model is *correct* or outputs an error message listing the detected misbehaviours. The final output of DeepLocalize contains the (1) fault type, (2) the layer and the phase (feed forward or backward propagation) in which the DL program has a problem, and (3) the iteration in which learning is stopped. The faults that the tool is able to detect include the following: “Error Before/After Activation”, “Error in Loss Function”, “Error Backward in Weight/$\varDelta$ Weight”, and “Model Does Not Learn” that suggests an incorrectly selected learning rate.

DeepDiagnosis (DD) (Wardat et al. 2022) was built on the basis of DeepLocalize and improved the latter by enlarging the list of detected symptoms and connecting them to a set of actionable suggestions. It detects ten types of faults: “Numerical Errors”, “Exploding Tensor”, “Unchanged Weight”, “Saturated Activation”, “Dead Node”, “Activation Function’s Output Out of Range”, “Loss Not Decreasing”, “Invalid Loss”, “Invalid Accuracy”, “Accuracy Not Increasing”, and “Vanishing Gradient”. Depending on the symptom, the actionable messages provided by DeepDiagnosis suggest to change either the loss or optimisation function, layer number, initialisation of weights, learning rate, or indicating that training data is “improper”. The authors perform an empirical evaluation of DeepDiagnosis and compare it to DeepLocalize, UMLAUT and AutoTrainer. They report the time overhead caused by each tool with respect to the number of faults it can successfully localise. However, the time overhead is not reported for each fault but for each dataset (i.e., for all faults of subject MNIST or Circle). In their empirical evaluation, the authors take the randomness associated with model training into account by running each of the compared tools 5 times.

As DeepLocalize does not provide an output that can be translated into a specific fault affecting the model, we only use DeepDiagnosis in the empirical comparison of fault localisation tools.

### UMLAUT

Similarly to DeepLocalize and DeepDiagnosis, UMLAUT (UM) (Schoop et al. 2021) operates through a callback attached to the model training call. This tool combines dynamic monitoring of the model behaviour during training with heuristic static checks of the model structure and its parameters. As an output, it provides the results of the checks along with best practices and suggestions on how to deal with the detected faults. The tool comprises ten heuristics for which the authors found mentions in different sources, such as API documentation and existing literature, lecture notes and textbooks, courses, blogs and non-scientific articles. The heuristics are divided by the area of application into “Data Preparation”, “Model Architecture” and “Parameter Tuning”. “Data Preparation” heuristics are dynamic and check if the training data contains “NaN”, has invalid shape, is not normalised or if the validation accuracy is higher than 95% after the third epoch of the training. On the other hand, all “Model Architecture” heuristics are static and are focused on the usage of correct activation functions. The “Parameter Tuning” category combines both dynamic and static rules that aim to detect over-fitting and control the values of the learning and dropout rates. As an output, the tool returns a list of heuristics that were violated. The empirical evaluation of UMLAUT was performed with 15 human participants and aimed mostly to determine whether it is useful for the developers. The authors considered only 6 bugs artificially injected across two DL systems. The reported results do not include the time the tool takes to run. Moreover, the authors do not account for the randomness associated with using the tool and do not compare it to any state-of-the-art tools.

### Neuralint

 Nikanjam et al. (2021) propose Neuralint (NL), a model-based fault detection approach that uses meta-modelling and graph transformations. The technique starts with building a meta-model for DL programs that consists of their base structure and fundamental properties. It then performs a verification process that applies 23 pre-defined rules, implemented as graph transformations, to the meta-model, to check for any potential inefficiencies. The rules are classified into four high-level root causes as suggested by Zhang et al. (2018): “Incorrect Model Parameter or Structure” (five rules), “Unaligned Tensor” (four rules), “API Misuse” (five rules), and “Structure Inefficiency” (nine rules). One example of “Unaligned Tensor” rule is a check whether consecutive layers in a model are compatible or whether the reshape of data did not lead to the loss of any elements. “API Misuse” includes a rule to check if the optimiser is correctly defined and connected to the computational graph. Another rule in this category inspects the parameter initialisation to detect the cases when initialisation is performed more than once or after the training has already started. The “Incorrect Model Parameter or Structure” rule checks if weights and biases are initialised with appropriate values and if suitable activation functions are used for specific layer types. “Structure Inefficiency” is responsible for detecting flaws in the design and structure of DNN that can result in the drop of model performance. Among others, there are rules in this category that check if the number of neurons in fully connected layers is decreasing when moving from the input to the output layer or rules that check that pooling layers are not used after each applied convolution, to avoid losing too much information about an input. The empirical evaluation of Neuralint does not include a comparison to any of the existing tools. The authors also do not report the time it takes to run the tool for each of the faults, but only provide information on the time for 5 selected DL models with different sizes.

### DeepFD

DeepFD  (Cao et al. 2022) (DFD) is a learning-based framework that leverages mutation testing and popular ML algorithms to construct a tool capable of labelling a given DL program as correct or faulty according to a list of common fault types the tool has learned to detect. To train the classifiers that lie at the core of the technique, the authors prepare a set of correct and faulty models. Faulty models are obtained through the artificial injection of up to five mutations to each correct program being used. The mutations that are used to inject faults are changing loss or optimisation function, changing learning rate, decreasing number of epochs, and changing activation functions. Consequently, these fault types correspond to the fault localisation capabilities of the tool. To construct the training dataset, all of the generated mutants and the original models are trained, while collecting run-time data of the same kind as for DeepLocalize, DeepDiagnosis, UMLAUT. The authors then extract 160 features from these data using statistical operations (e.g., calculating skewness, variance or standard deviation). As the next step, three popular ML algorithms (K-Nearest Neighbors Altman 1992, Decision Tree Breiman 2017 and Random Forest Ho 1995) are trained on the created dataset. A union of the prediction results of these classifiers is used for fault localisation in a given program under test. DeepFD outputs a list of detected faults along with the code lines affected by each fault type. The empirical evaluation of DeepFD contains comparison to AutoTrainer and DeepLocalize. The authors take the stochasticity of the proposed approach into account and run their experiments 10 times. However, no information is reported on the time required to train and run DeepFD.

### Autotrainer

AutoTrainer (Zhang et al. 2021) is an automated tool whose aim is to detect, localise, and repair training related problems in DL models. It starts with an already trained and saved underperforming DL model. To check the model, AutoTrainer continues the training process and observes different internal parameters such as loss values, accuracy, gradients, etc. The collected information is then analysed and verified against a set of rules that are aimed to detect potential training failures. In particular, the authors focus on the symptoms of exploding and vanishing gradients, oscillating loss, slow convergence and ‘dying ReLU’. To deal with each of the reveled symptom, AutoTrainer applies an ordered set of possible solutions to the model. After a potential solution is applied, the tool continues training the model for one more epoch to check whether the symptom is gone or not. The solutions include changing activation functions, hyperparameters such as learning rate and batch size, optimisers and weight initialisation, and addition of batch normalisation layers to the model structure. We do not consider AutoTrainer in this empirical comparison as its final goal is to patch an already trained model rather than localise and fix the source of a DNN’s misbehaviour.

The tools described in this section are built using a limited set of rules and best practices, fixed thresholds or training data, resulting in an urgent need to empirically investigate the generalisability of these approaches to diverse programs and architectures. While some of the experiments conducted by the proponents to evaluate these tools include comparisons to other existing tools, no work considers the full set of four existing FL approaches. Moreover, the existing evaluations do not always consider the randomness associated with the training of the DL models and do not report detailed information on the runtime costs associated with the FL tools. Most importantly, no third party evaluation of these tools on a curated dataset of faulty DL models was ever conducted and reported so far.

## Benchmark

To evaluate and compare the fault localisation techniques selected for this study, we adopt a carefully selected benchmark of faulty models from the existing literature (Kim et al. 2023). This benchmark is of a particular interest as it combines both models affected by real-world faults and those deliberately produced using artificial faults.

### Fault Types and Tool Coverage

In Table 1, we introduce abbreviations (column ‘Abbrev.’) for all the ground truth fault types encountered in the benchmark (see Tables 2 and 3) or suggested in the output of the evaluated FL tools. Most of the abbreviations (except for BCI and CPP) are adopted from the work that proposes mutation operators for DNNs derived from real faults (Humbatova et al. 2021). Column ‘Freq.’ (frequency) indicates how many times a specific fault type is encountered in the benchmark, while columns with tool names show whether each tool can detect a specific fault type. The column ‘Cov.’ (coverage) shows how many tools can localise each fault type. We separate the fault types obtained from the evaluation benchmark from those that were extracted from the output of the FL tools after their execution on the benchmark. The separating row (starts with ’Count’) provides the number of fault types in the benchmark (under ‘Fault Type’ column), total number of ground truth faults (under ‘Freq.’ column), and the number of covered fault types for each tool. In the last row, we provide total numbers when taking the fault types coming from tool outputs into account.

**Table 1:** Fault types and abbreviations. Coverage of fault types by FL tools

Abbrev.	Fault Type	Freq.	DFD	DD	NL	UM	Cov.
HBS	Wrong batch size	4	N	N	N	N	0
HLR	Wrong learning rate	6	Y	Y	N	Y	3
HNE	Change number of epochs	7	Y	N	N	N	1
ACH	Change activation function	13	Y	Y	Y	Y	4
RAW	Redundant weights regularisation	1	N	N	N	N	0
WCI	Wrong weights initialisation	3	N	Y	Y	N	2
LCH	Wrong loss function	7	Y	Y	Y	N	3
OCH	Wrong optimisation function	5	Y	Y	Y	N	3
LRM	Missing layer	1	N	Y	Y	Y	3
LCN	Wrong number of neurons in a layer	2	N	N	Y	N	1
VRM	Missing validation set	1	N	N	N	N	0
CPP	Wrong data preprocessing	3	N	Y	N	Y	2
Count	12	53	5	7	6	4	−
LAD	Redundant layer	0	N	Y	Y	N	2
LCF	Wrong filter size in a convolutional layer	0	N	N	Y	N	1
BCI	Wrong bias initialisation	0	N	N	Y	N	1
IWS	Wrong shape of input data	0	N	N	N	Y	1
Total	16	53	5	8	9	5	−

**Table 2:** Evaluation benchmark, artificial faults: the Fault Type can be real (R) or artificial (A); the fault Id identifies subject MNIST mutants (M), CIFAR mutants (C), Reuters mutants(R), Udacity mutants (U), and Speaker Recongnition (S); Source shows the dataset of origin; the models are divided into two groups of classification (C) or regression (R) task

Fault Type	Id	SO Post #	Source	Task	Faults
		/Subject			
A	M1	MN	DeepCrime	C	Wrong weights initialisation (0)
A	M2	MN	DeepCrime	C	Wrong activation function (7)
A	M3	MN	DeepCrime	C	Wrong learning rate
A	C1	CF10	DeepCrime	C	Wrong activation function (2)
A	C2	CF10	DeepCrime	C	Wrong number of epochs
A	C3	CF10	DeepCrime	C	Wrong weights initialisation (2)
A	R1	RT	DeepCrime	C	Wrong weights regularisation (0)
A	R2	RT	DeepCrime	C	Wrong activation function (2)
A	R3	RT	DeepCrime	C	Wrong learning rate
A	R4	RT	DeepCrime	C	Wrong loss function
A	R5	RT	DeepCrime	C	Wrong optimiser
A	R6	RT	DeepCrime	C	Wrong weights initialisation (0)
A	R7	RT	DeepCrime	C	Wrong activation function (2)
A	U1	UD	DeepCrime	R	Wrong loss function
A	U2	UD	DeepCrime	R	Wrong optimiser
A	S1	SR	DeepCrime	C	Wrong loss function
A	S2	SR	DeepCrime	C	Wrong number of epochs

The authors of DeepFD identified five most frequent types of faults in the benchmark they used for evaluation and designed their tool to detect these specific fault types. In particular, they cover HLR, ACH, LCH, HNE and OCH fault types. Despite being a small fraction of all the recognised faults that affect DL systems, these fault types are frequently encountered in the real world (Humbatova et al. 2020) as well as in our benchmark. By observing how the internal variables of a DNN change during the training process, DeepDiagnosis is designed to detect 10 different fault symptoms, such as vanishing gradients or numerical errors. It maps these symptoms to 7 different fault types. Similarly to DeepFD, it can detect LCH, ACH, HLR, OCH faults, and additionally, DeepDiagnosis can detect the WCI fault, as well as problems in the training data and in the number of layers. In the last case, DeepDiagnosis ’s ‘Change the layer number’ corresponds to two distinct fault types in Table 1, i.e., ‘Missing layer’ and ‘Redundant layer’. Correspondingly, DeepDiagnosis covers 8 fault types encountered in this study (7 from the benchmark). UMLAUT operates based on a set of both dynamic and static checks performed before and during training of a model. The used heuristics cover hyperparameter tuning, and while problems with learning rate are detected also by the previously discussed approaches, UMLAUT can also detect high drop out rate, while it does not pay attention to the number of epochs as DeepFD  (Schoop et al. 2021). When it comes to the fault types encountered in our benchmark, UMLAUT can only detect 4 out of 12. Similarly to DeepDiagnosis, UMLAUT can detect problems with training data and its pre-processing and also covers problems with activation functions of the model. Neuralint relies on meta-modelling and graph transformations to perform a verification process based on 23 pre-defined rules that cover the initialisation of different parameters, nuances of neural network architecture, and API misuse (Nikanjam et al. 2021). The detection of the violation of these rules leads to a number of diverse recommended fixes that cover data pre-processing, selection of optimiser, activation functions and tuning of the neural network architecture. As a result, Neuralint covers the largest number of fault types (9 out of 16). However, it covers only 6 fault types of the benchmark as opposed to 7 of DeepDiagnosis.

The observations show that 3 fault types (HBS, RAW, and VRM) are not covered by any of the considered tools, while ACH is the only one that is covered by all FL approaches. The remaining fault types are covered by 1 (HNE, LCN, LCF, BCI, IWC), 2 (WCI, LAD, CPP) or 3 (HLR, LCH, OCH, LRM) of the tools. These findings show that despite some similarities in the types of detected faults, all of the approaches have their own specifics and vary in the localisation methods used. At the current state of the art, it appears that the available approaches are rather complementary to each other.

### Evaluation Benchmark

*Artificial faults* of our benchmark were produced by DeepCrime  (Humbatova et al. 2021), a state-of-the-art mutation testing tool for DL systems based on real faults (Humbatova et al. 2020). The subject models in DeepCrime ’s mutants dataset cover a diverse range of application areas, such as handwritten digit classification (MN), speaker recognition (SR), self-driving car designed for the Udacity simulator (UD), eye gaze prediction (UE), image recognition (CF10), and categorisation of news articles (RT). Kim et al. (2023) selected 25 most representative faults from this dataset for the evaluation of DNN repair approaches. These faults are generated by injecting 9 distinct fault types into originally well-performing models. As our initial experimentation showed, all 4 evaluated FL tools crash on 8 UE faults because this subject uses a more complex, Functional type of Keras’s DNN, and a custom loss function. We adopted the remaining 17 faults for the needs of this study.

The benchmark compiled by Kim et al. (2023) also contains a number of *real faults*. This section of their benchmark was derived from the set of issues that were collected and used for the evaluation of the DeepFD tool (Cao et al. 2022, 2021). This initial set contains 58 faulty DNNs collected from bug-reporting platforms such as StackOverflow and Github. Consequently, Kim et al. (2023) analysed this benchmark by performing a series of checks. Specifically, they investigated the reproducibility of faults from the DeepFD benchmark, checking if the reported faulty model, its training dataset, the fault, and its fix accurately correspond to the source of the real fault (i.e., the original StackOverflow post or GitHub commit). In addition, they analysed if the fault can be in fact reproduced, i.e., the faulty model demonstrates performance worse than that of the fixed model and both are executable. If these conditions were not satisfied, such a fault was deemed non-reproducible and unsuitable for evaluation. As a result, only 9 out of 58 issues proved to be reliably reproducible.

To expand the pool of real faults, we refer to an empirical study by Jahangirova et al. (2024) where the authors analyse all available repositories of real DNN faults in a systematic way. In particular, they perform a manual analysis of 490 faults from five benchmarks: TFBugs2018 (Zhang et al. 2018), DeepLocalize  (Wardat et al. 2021), DeepFD  (Cao et al. 2022), Defects4ML (Morovati et al. 2023), and SFData (Wu et al. 2021, 2022). Their findings show that 176 of these faults are invalid, primarily due to missing links to the source (bug report), absence of code or fixes, or misinterpretation of the source fault report. When analysing the remaining 314 faults, the authors assess their realism using 4 specific criteria: (1) the source code for the buggy version in the benchmark must match with the buggy code described in its original source (e.g., a bug report); (2) the fix implemented in the benchmark must correspond to the fix detailed in the source; (3) the training data used in the benchmark must be consistent with that specified in the source; (4) the training data should be realistic, meaning it should either reference well-known existing datasets, be collected through a rigorous process, or be generated using a robust mathematical or statistical method (Jahangirova et al. 2024). The results indicate that only 58 faults out of 314 satisfy all four realism criteria. They further evaluate the reproducibility of these 58 faults, finding that only 18 are reproducible and stable, consistently exhibiting faulty and fixed behaviour across 20 runs. However, 5 out of these 18 faults are unusable due to program crashes, and another 5 were duplicates of previously considered faults. Moreover, for one specific fault, all our FL tools are not applicable. As a result, we could successfully complement the benchmark of Kim et al. (2023) by adding 7 new real faults, making it 16 in total.

**Table 3:** Evaluation benchmark, real faults: the Fault Type can be real (R) or artificial (A); Source shows the dataset of origin; SO Post # / Subject shows ID from the dataset of origin; the models are divided into two groups of classification (C) or regression (R) task

Fault	Id	SO Post #	Source	Task	Faults
Type		/Subject			
R	D1	31880720	DeepFD	C	Wrong activation function (7)
R	D2	41600519	DeepFD	C	Wrong optimiser | Wrong batch size
					Wrong number of epochs
R	D3	45442843	DeepFD	C	Wrong optimiser | Wrong loss function
					Wrong batch size | Wrong activation function (0,1)
					Wrong number of epochs
R	D4	48385830	DeepFD	C	Wrong activation function (0,1)
					Wrong loss function | Wrong learning rate
R	D5	48594888	DeepFD	C	Wrong number of epochs | Wrong batch size
R	D6	50306988	DeepFD	C	Wrong learning rate | Wrong number of epochs
					Wrong loss function | Wrong activation function (1)
R	D7	51181393	DeepFD	R	Wrong learning rate
R	D8	56380303	DeepFD	C	Wrong optimiser | Wrong learning rate
R	D9	59325381	DeepFD	C	Wrong data preprocessing
					Wrong activation function (5,6) | Wrong batch size
R	D10	024	Defect4ML	R	Wrong optmiser | Wrong number of epochs
					Missing validation set
R	D11	068	Defect4ML	C	Wrong activation function (7)
R	D12	098	Defect4ML	C	Wrong data preprocessing
R	D13	099	Defect4ML	C	Missing layer | Wrong number of neurons (0)
					Wrong activation function (1)
R	D14	48221692	DeepLocalize	R	Wrong activation function (1)
R	D15	50079585	DeepLocalize	C	Wrong number of neurons (13) | Wrong loss function
					Wrong activation function (14)
					Wrong data preprocessing
R	D16	kerasma	DeepLocalize	C	Wrong number of neurons (1)

The complete benchmark used in this study can be found in Tables 2 and 3. Column ‘Fault Type’ shows whether the fault was real (‘R’) or artificially seeded (‘A’). Column ‘Source’ shows the fault’s parent dataset. Column ‘Id’ bears the ID of the fault which will be reused throughout the paper. These IDs refer to real faults curated from the DeepFD, DeepLocalize, and Defect4ML datasets using a prefix D followed by a number from 1 to 16. For artificial faults, we use the first letter of the corresponding subject name (i.e. M for MNIST of C for CIFAR-10). Column ‘SO Post #/Subject’ provides the StackOverflow post number for issues in DeepFD and DeepLocalize, as obtained from the StackOverflow platform, or the the subject name, in the case of artificial faults and GitHub issues in the DeepLocalize dataset, and finally, ID of the fault, when taken from Defect4ML dataset. Column ‘Task’ has ‘C’ for faults that solve a classification problem and ‘R’ for those dealing with a regression task. Finally, column ‘Faults’ contains the ground truth associated with faults. Where applicable, the fault description is accompanied by a set of layer identifiers in parentheses, specifying the layers affected by the fault. For example, ‘Wrong activation function (1, 3)’ indicates that the activation function should be changed in layers 1 and 3 to fix the fault.

## Neutrality Analysis

In the context of fault localisation in DNNs, our goal is to identify and localise faults in the network architecture and hyperparameters. Even if there are architectures widely used for specific tasks (e.g., LeNet-5 for handwritten character recognition LeCun et al. 2020), there are no strict rules that dictate a single optimal architecture with specific hyperparameter values. Although the benchmarks that we use in this study include the ground truth (GT) for faulty models, i.e., repaired models for real faults, and original and un-mutated versions for artificial faults, there is often not only one possible way to ‘fix’ a model when a fault is identified. This suggests the potential for discovering alternative patches that not only complement the known patch (i.e., GT) by suggesting different ways of repairing but also possibly exhibit better performance than the known one. Identifying such alternative patches would enable a more precise and fair evaluation of FL techniques that could produce valid outputs different from the known GT.

In our search for alternative patches, we are inspired by the notion of software neutrality, which states that a random mutation of an executable program is considered *neutral* if the behaviour of the program on the test set does not change (Renzullo et al. 2018). This neutrality analysis aims to investigate diverse patches with similar or better fitness: these can be utilised as alternative Ground Truths. Since our targets are DL programs, the conditions for performing a neutrality analysis differ from those of traditional programs. For example, fitness is now measured by model performance with standard metrics such as test set accuracy. This means that fitness evaluation involves training and testing the model. Moreover, during fitness evaluation it is important to account for the inherent stochastic properties of DL programs because the model’s performance can vary with multiple trainings. To address this, in our algorithm below, we train the model ten times and calculate the fitness as an average of the resulting ten accuracy values.

**Algorithm 1:**
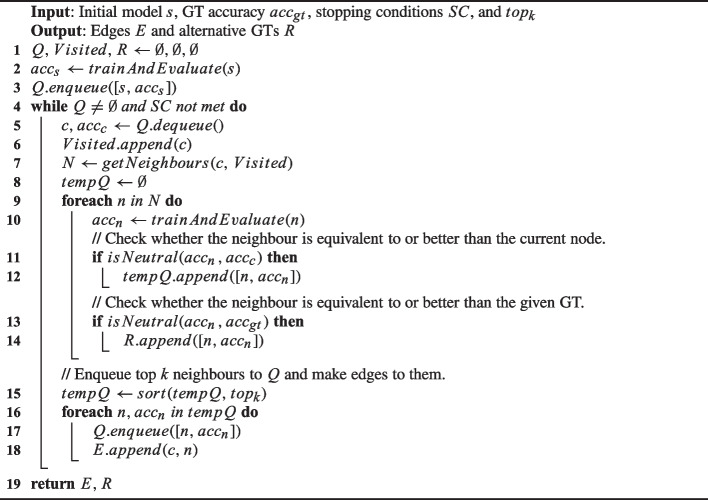
Breadth-First Search (BFS) for neutrality analysis.

**Table 4:** Mutation operator for neutrality analysis

Operator	Description
Change activation function	It replaces the value of the hyperparameter of the given model with other pre-defined values from Keras.
Change kernel initialisers	
Change bias initialisers	
Change loss function	
Change optimiser	
Change learning rate	It changes the learning rate by either multiplying it by ten or dividing it by ten.
Change epochs	It changes the epochs by either multiplying it by two or dividing it by two.
Change batch size	It replaces the batch size with other pre-defined values of 16, 32, 64, 128, 256, 512.
Change layer	It either duplicates or deletes the given layer.
Change the number of neurons in a given dense layer	It changes the number of neurons by either multiplying its number by two or dividing it by two.

Algorithm 1 presents the Breath-First Search (BFS) for our neutrality analysis on DL programs. This algorithm takes as inputs an initial (buggy) model *s*, the accuracy of the known GT $acc_{gt}$, and stopping criteria *SC*. The outputs are a list of alternative GTs and edges of the neutrality graph. The algorithm starts with training and evaluating the initial buggy model before putting it in the queue (Lines 2-3). Next, it begins a search loop where it iteratively retrieves a model (i.e., a parent model *c*) along with its accuracy $acc_{c}$ from the queue (Line 5). Subsequently, the algorithm explores all adjacent models (i.e., neighbours) that are obtained by applying a distinct single mutation on *c* (Line 7). Each mutation involves changing a single hyperparameter of the model, in other words, neighbouring models differ from their parent model by one hyperparameter. The details of mutation operators adopted from Kim et al. (2023) are shown in Table 4. Then, the algorithm iterates over the neighbours by training and evaluating them (Line 10), and evaluates the *neutrality*^1^ of each neighbour compared to the parent model (Line 11) and the known GT (Line 13). Since sometimes the number of neutral neighbours is numerous, potentially impeding the exploitation of the search, the algorithm places them into the temporal queue, not in the main queue (Line 12). If it is neutral with respect to the known GT, it is added to a list of alternative GTs (Line 14). After this iteration, the algorithm sorts the temporal queue by accuracy and takes only top-k performing neighbours by enqueueing them into the main queue. The search process stops when it meets the given stopping criteria or the queue is empty. As the algorithm evolves the model by applying mutation to its parent, the resulting alternate GTs are usually higher-order mutants of the initial buggy model.

**Fig. 1:**
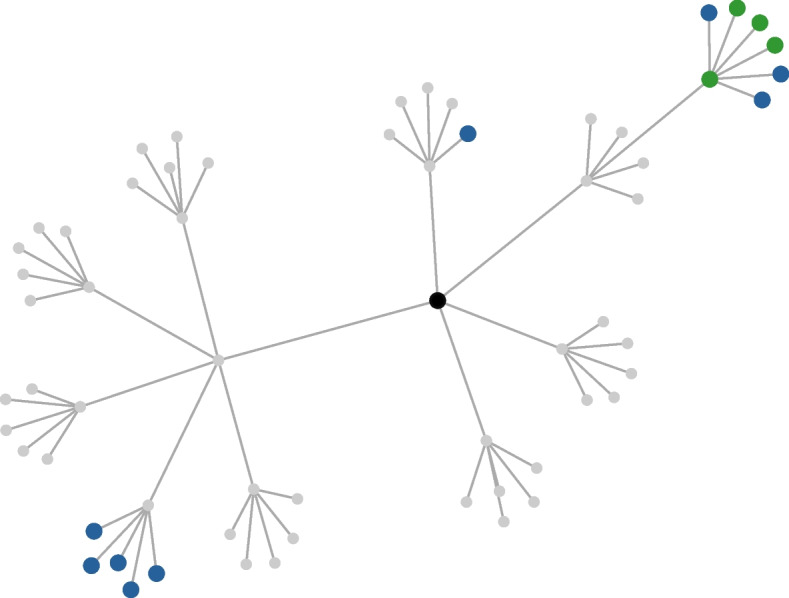
An example neutrality network of D4

Based on the search results, we can draw a so-called neutrality graph or ‘network’ (Renzullo et al. 2018), as shown in Fig. 1. Each edge represents a single mutation and each node represents the DL models (i.e., mutants). A black node denotes the initial buggy model and other nodes are neutral with their parent node. Among them, the ones that are on par with or better than the known patch are coloured in either blue or green. In particular, models that outperform the known GT with statistical significance are marked blue.^2^ Those that are not statistically significant but exceed the known patch in terms of average accuracy are coloured in green. In this example, we found 13 alternative patches that fix the buggy model differently but show equal or higher performance than the known patch.

## Empirical Study

### Research Questions

The *aim* of this empirical study is to compare existing DL fault localisation approaches and to explore their generalisability to different subjects represented by our benchmark of artificial and real faults. To cover these objectives, we define the following three research questions:

- **RQ1. Effectiveness**: *Can existing FL approaches identify and locate defects correctly in faulty DL models? How do the outcomes differ when considering alternative GTs?*
- **RQ2. Stability**: *Is the outcome of fault identification analysis stable across several runs?*
- **RQ3. Efficiency**: *How costly are FL tools when compared to each other?*

RQ1 is the key research question for this empirical study, as it evaluates the effectiveness of different FL tools on our curated benchmark of artificial and real faults. By addressing this question, our study aims to provide a deeper understanding of the practical utility of FL tools across a range of diverse scenarios. RQ1 is further divided into two sub-questions to address the alternative GTs: RQ1.1 focuses on the original GT, while RQ1.2 considers all alternative GTs resulting from the neutrality analysis. Note that while these tools were originally evaluated in their respective studies, disparate datasets and metrics were used for each tool. Our goal is to provide a unified evaluation by applying all the tools to the same benchmark.

RQ2 focuses on the fact that the output of the FL tools can be affected by randomness and therefore there is a possibility of getting different results at different runs. If a simple rerun of the tool produces a completely different outcome, then its reliability for practical use becomes questionable. As part of this research question, we run the tools multiple times and report how stable the outcomes are. By quantifying the extent of variability in the results across diverse sets of faults, we aim to produce insight into the reliability and robustness of the evaluated tools.

The main goal of FL tools is to assist developers in identifying faults in software systems. However, if this support comes at a high price, i.e., one run of an FL tool takes a long time to complete, the usefulness of such tools and the feasibility of using them becomes questionable. RQ3 examines this dimension and reports the time it takes to run each tool and how these times vary across the tools. This analysis not only highlights the computational efficiency of the tools but also provides insights into how performance trade-offs might influence their adoption in practice.

#### Processing Tool Output

The output formats of the DL tools differ depending on the technique. For example, DeepFD generates a list of detected fault types in the given DL program, and it can also indicate the line numbers where faults are localised. In contrast, UMLAUT produces warnings and critical messages at the end of each training epoch, often limited to a few words or a brief sentence. DeepDiagnosis, similar to UMLAUT, uses a tool-specific callback to monitor the training process, but stops training and writes any identified faults to a file once a symptom is detected. These faults are localised to specific layers, and the tool often offers fault types and potential fixes where possible. The output tends to be brief, typically limited to a short sentence per component (e.g., symptom, fault type, or fix). On the other hand, Neuralint can link faults either to specific layers or to a general ‘Learner’ function, presenting detailed fault descriptions with one or two sentences. In our experiments, one of the authors manually analysed all outputs and mapped them to the corresponding fault types, adding new types as needed.

### Experimental Settings & Evaluation Metrics

For the comparison, we adopt publicly available versions of all considered tools (Cao et al. 2021; Schoop et al. 2021; Nikanjam et al. 2021; Wardat et al. 2021) that are run on Python with library versions specified in the requirements for each tool. However, we had to limit the artificial faults to those obtained using CF10, MN, and RT as DeepDiagnosis is not applicable to other subjects.

The authors of DeepFD adopted the notion of statistical mutation killing (Jahangirova and Tonella 2020) in their tool. They run each of the models used to train the classifier as well as the model under test 20 times to collect the run-time features. For fault localisation using DeepFD, we adopt an ensemble of already trained classifiers provided in the tool’s replication package. Similar to the authors, for each faulty model in our benchmark, we collect the run-time behavioural features from 20 retrainings of the model. Neuralint is based on static checks that do not require any training and thus, are not prone to randomness. We run each of the remaining tools 20 times to account for the randomness in the training process and report the most frequently observed result (mode). Our experiments of running FL tools were conducted on machines with an AMD Ryzen 7 3700X 8-Core CPU, Nvidia GeForce RTX 2060 GPU, and 32GB of RAM, operating on Windows 11 Pro.

To calculate the similarity between the ground truth provided for each fault in our benchmark and the fault localisation results, we adopt the standard information retrieval metrics Precision (PR), Recall (RC) and $F_{\beta }$ score:

1 $$\begin{aligned} RC = \frac{| FT_{loc} \cap FT_{gt} |}{| FT_{gt} |} \end{aligned}$$

2 $$\begin{aligned} PR = \frac{| FT_{loc} \cap FT_{gt} |}{| FT_{loc} |} \end{aligned}$$

3 $$\begin{aligned} F_{\beta } = (1 + \beta ^2) \frac{PR \cdot RC}{\beta ^2 PR + RC} \end{aligned}$$

Recall measures the proportion of correctly reported fault types in the list of localised faults ($FT_{loc}$) among those in the ground truth ($FT_{gt}$); Precision measures the proportion of correctly reported fault types among the localised ones; $F_{\beta }$ is a weighted geometric average of *PR* and *RC*, with the weight $\beta$ deciding on the relative importance between *RC* and *PR*. Specifically, we adopt $F_{\beta }$ with $\beta$ equals 3, which gives three times more importance to recall than to precision. This choice of beta is based on the assumption that in the task of fault localisation, the ability of the tool to find as many correct fault sources as possible is more important than the precision of the answer.

For neutrality analysis, we set $top_k$ to 5 and the stopping condition *SC* to a 48-hour time budget. During the search, every model is trained ten times and we use a mean of the ten metric values depending on the task solved by each subjects (i.e., accuracy for classification or loss for regression).

For the efficiency analysis, we measure the runtime of the tools in seconds and perform Wilcoxon rank sum test on the acquired results.

**Table 5:** Ground Truth (GT) and fault localisation outcome generated by DeepFD (DFD); #F indicates the number of ground truth faults, while #M the number of ground truth faults detected by the tool (with underline used to indicate the best result among all tools being compared). Avg. shows the average within artificial or real faults. T.A. shows the total average across faults

	

## Results

### RQ1.1 (Effectiveness Before Neutrality Analysis)

Tables 5, 6, 7, and 8 present the output of the application of fault localisation tools (DFD, DD, NL, and UM, respectively) to our benchmark. Column *‘GT’* stands for *‘Ground Truth’* and provides the list of fault types affecting the model, while column ‘*#F*’ reports the length of this list. Column ‘${<tool\_name>-output}$’ contains the fault list generated by each FL tool, while column *‘Matches-GT’* indicates for each fault in the ground truth whether it was detected by the tool or not (1 if yes and 0 otherwise) and column *‘#M’* counts the number of ground truth faults detected by a corresponding tool. For each row (issue) we underline the number of detected faults (‘$\#M$’) if the tool was able to achieve the best result across all the compared approaches. We provide the average number of fault types detected for issues generated by artificially injected faults or real-world issues (rows ‘Avg.’) and across the whole benchmark (row ‘T.A.’, i.e., Total Average).

**Table 6:** Ground Truth (GT) and fault localisation outcome generated by DeepDiagnosis (DD); #F indicates the number of ground truth faults, while #M the number of ground truth faults detected by the tool (with underline used to indicate the best result among all tools being compared). Avg. shows the average within artificial or real faults. T.A. shows the total average across faults

**Table 7:** Ground Truth (GT) and fault localisation outcome generated by Neuralint (NL); #F indicates the number of ground truth faults, while #M the number of ground truth faults detected by the tool (with underline used to indicate the best result among all tools being compared). Avg. shows the average within artificial or real faults. T.A. shows the total average across faults

	

When faults affect only selected layers, we specify the indexes of the faulty layers within brackets, for ground truth and for fault localisation results, if this information is provided. Moreover, ‘-’ means that an FL tool was not able to find any fault in the model under test; ‘N/A’ means that the tool was not applicable to the fault type in question or crashed on it. For example, Neuralint accepts only optimisers that are defined as strings (e.g., ‘sgd’), which in turn implies that the default learning rate as defined by the framework is used. This makes it not possible for Neuralint to find an optimiser with modified learning rate. Symbol ‘,’ separates all detected faults, while separation by ‘|’ means that the faults are alternative to each other, i.e., the tool suggests either of them could be the possible cause of model’s misbehaviour.

**Table 8:** Ground Truth (GT) and fault localisation outcome generated by UMLAUT (UM); #F indicates the number of ground truth faults, while #M the number of ground truth faults detected by the tool (with underline used to indicate the best result among all tools being compared). Avg. shows the average within artificial or real faults. T.A. shows the total average across faults

**Table 9:** Number of Ground Truth (GT) faults (#F); Recall (RC), Precision (PR) and $F_3$ measure for each FL tool. Avg. shows the average within artificial or real faults. T.A. shows the total average across faults

	

Interestingly, for most of the applicable faulty models UMLAUT (31 out of 33) and DeepDiagnosis (19 out of 28) suggest changing the activation function of the last layer to ‘softmax’ even if in 61% of these cases for UMLAUT and 54% for DeepDiagnosis, the activation function is already ‘softmax’. This also happens once to Neuralint. We exclude such misleading suggestions from the tools’ output. Moreover, sometimes UMLAUT mentions that overfitting is possible. Since it is just a possibility and such a message does not point to a specific fault, we also exclude it from our analysis. The complete output messages provided by the tools are available in our replication package (Humbatova et al. 2023).

Table 9 reflects the overall evaluation of the effectiveness of the FL tools. Column ‘$GT \# F$’ shows the number of fault types in the ground truth, while columns ‘${<tool\_name>}$’ contain all measured performance metrics for each tool: columns ‘*RC*’ report the values of Recall, columns ‘*PR*’ report Precision, and columns ‘$F_3$’ the $F_\beta$ score with $\beta = 3$. We use the $F_3$ metric because in our context recall is very important, as it directly reflects the ability of the tool to identify all relevant faults. While precision also remains valuable, it is less of a priority compared to ensuring high recall. The $F_3$ score, by assigning recall a weight three times greater than precision, balances these considerations effectively. It ensures that significant penalties are incurred for missing relevant faults while still maintaining sensitivity to precision. Using $F_2$, which weights recall only twice as much as precision, might under-emphasise recall, leading to suboptimal evaluation of the tools’ fault localisation capabilities. In contrast, $F_4$ may over-emphasise recall to the extent that precision is insufficiently considered, potentially resulting in too many false positives that the user has to investigate. By selecting $F_3$, we strike an optimal balance that prioritises recall strongly while still valuing precision to a meaningful degree. The results for $F_1$ and $F_2$ are available in the replication package (Humbatova et al. 2023) and are generally aligned with those obtained for $F_3$, i.e. all conclusions about the performance comparison of tools hold, disregarding the selection of specific values for $\beta$.

We treated the cases when a tool is not applicable to an issue as if the tool has failed to locate any faults affecting the issue. We provide mean values for each tool across artificial and real faults (rows ‘Avg.’) and across all issues in the benchmark (row ‘T.A’), to ease the comparison between the tools. According to these numbers, DeepFD, on average, exhibits the best performance and significantly outperforms other tools on real faults. This can be influenced by the fact that the ‘Real Fault’ part of the benchmark partly comes from the evaluation benchmark of DeepFD, as this was one of the few available sources of truly reproducible real faults. The selection of the fault types that DeepFD is trained to detect was influenced by the distribution of faults in the evaluation benchmark, as described in the corresponding article (Cao et al. 2022). For artificial faults, DeepFD, Neuralint, and UMLAUT achieve equal RC performance, higher than that of DeepDiagnosis, with Neuralint, DeepDiagnosis and UMLAUT having higher PR and $F_3$ score than DeepFD. Overall, based on all the measured metrics, DeepFD has the highest RC values, while DeepDiagnosis ’s RC measurements are noticeably lower than for other tools. Neuralint and UMLAUT show similar performance according to the RC metric, while PR is slightly higher for Neuralint (0.23 vs 0.20), which achieved the highest values across all the considered tools.

Despite the inferior performance of DeepDiagnosis in our experiments, the authors of DeepDiagnosis achieved higher performance for their tool than UMLAUT in their evaluation. They used 2 separate sets of faulty programs. According to the results, DeepDiagnosis could correctly identify 87% of the buggy models from one benchmark and 68% from another, while UMLAUT was only able to identify 49% and 35% of faulty models from these benchmarks, respectively. UMLAUT ’s authors, in their turn, did not perform any empirical comparison with existing FL tools, and instead carried out a human study to measure how useful and usable is their tool for developers that aim to find and fix bugs in ML programs (Schoop et al. 2021). The authors of Neuralint also did not perform any comparison, but they have evaluated the performance of their tool on a set of 34 real-world faulty models gathered from SO posts and Github (Nikanjam et al. 2021). Their evaluation showed that Neuralint was able to correctly detect 71% of all the faults found in these issues. The authors of DeepFD have performed their evaluation on a benchmark consisting of 58 real-world faults (Cao et al. 2022), that were later analysed by the authors of the benchmark (Kim et al. 2023) that we included in the real fault section for our study. Their evaluation showed that DeepFD can correctly localise 42% of the ground truth faults observed in their benchmark, while UMLAUT could only detect 23%.

**Table 10:** Neutrality Analysis

	

It is worth mentioning that, unlike other tools, DeepFD does not provide layer index suggestions. Thus, it is not possible to understand whether a successfully detected fault of ‘ACH’ type actually points to the correct layer. This is the case for five of the 33 issues and if we exclude these issues from the calculation of the average RC, the result for DeepFD drops from 0.33 to 0.20, which makes it comparable to Neuralint (0.19) and UMLAUT (0.19). If we assume that DeepFD correctly locates this fault with the probability of 50% (the suggested layer is either correct or not), the mean RC value will be equal to 0.27. Also, for some of the fault types, other tools, but DeepFD provide specific suggestions on which activation function (DD, UM) or weights initialisation (NL) to adopt or whether to increase or decrease the learning rate (UM).

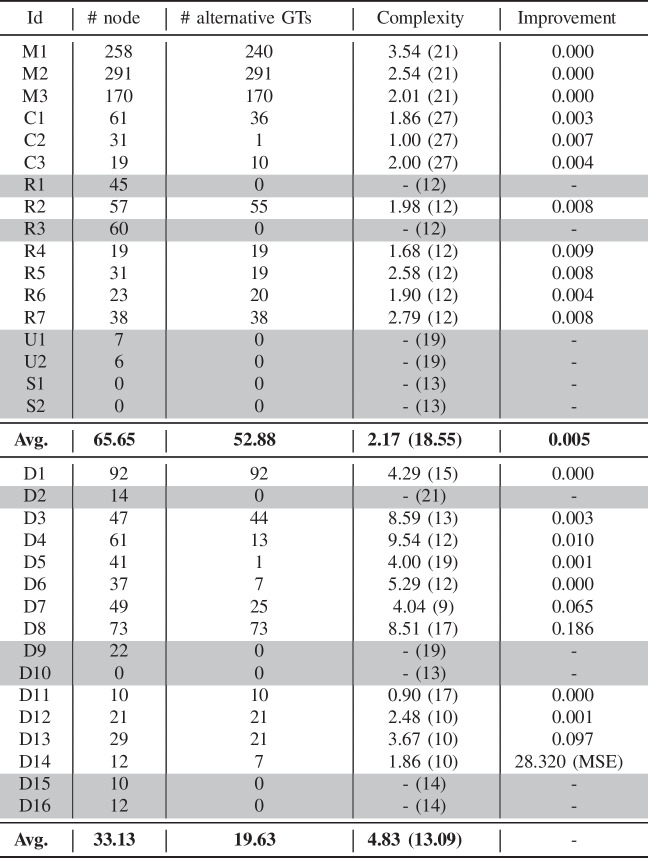



### RQ1.2 (Effectiveness after neutrality analysis)

We have subsequently investigated our hypothesis that relying on a single ground truth, represented by a single set of changes that improve the model performance, may not be sufficient.

Table 10 shows the number of nodes in the neutrality graph, all of which are neutral relative to their parent nodes, and the number of found alternative GTs that achieve equal or better performance than the known GT. For faulty models that lack alternative ground truths, the corresponding results are shaded grey. Column ‘Complexity’ shows how much the alternative GTs differ from the known GT, measured by counting how many hyperparameters differ between them. For each row, we calculate the complexity averaged over all found alternative GTs. The number in brackets indicates the total number of hyperparameters for each fault. Column ‘Improvement’ shows the extent of performance improvement over the known GT in terms of the evaluation metric (e.g., accuracy), measured as the average difference across all alternative GTs found. For 11 faults (R1, R3, U1, U2, S1, S2, D2, D9, D10, D15, D16), it was not possible to identify alternative patches within the available budget, and therefore no results could be calculated (marked with ‘-’). The number of nodes varies depending on the dataset/model, with relatively smaller models such as MNIST (M1, M2, and M3) producing a more expanded network than others.

Through our neutrality analysis, we identify an average of 53 alternative GTs for artificial faults and 20 for real faults, revealing the existence of alternative GTs and could impact the evaluation of fault localisation tools. Typically, the complexity of real faults (4.83 on average) is higher than artificial faults (2.17 on average). This may stem from the fact that artificial faults are simpler by definition: by construction, only one hyperparameter is mutated, compared to the GT, whereas real faults tend to be more complex. In terms of performance improvement of the alternative GTs over the known GT, we observe that there are only marginal improvements, although the improvements are more pronounced for real faults compared to artificial faults. This could be attributed to the fact that the answers obtained from StackOverflow are not always ideal and may sometimes only suggest a partial fix.

Based on the results of neutrality analysis, we have recalculated the fault localisation results for all the tools evaluated using the RC, PR or $F_3$ score. Table 11 shows results for each tool and issue that can be observed when using all the alternative ground truths, in addition to the original one. For issues where it was not possible to locate alternative ground truths, the results are greyed out. For issues on which a tool improved its performance after neutrality analysis, we indicate the improved RC, PR and $F_3$ score in boldface. The PR values that have decreased as a result of taking alternative ground truth into account are underlined. In this table, we report the maximum RC observed across all ground truth variants and the average PR and $F_3$ calculated on these GTs. To simplify the comparison of the tools before and after neutrality analysis, in Table 12 we provide initial average RC, PR and $F_3$ scores, along with the new ones, for the two benchmark sections (AF denotes artificial faults, RF real faults) and overall (T.A.: Total Average).

**Table 11:** Recall (RC), Precision (PR) and $F_3$ measure for each FL tool after neutrality analysis. Avg. shows the average within artificial or real faults. T.A. shows the total average across faults. The values that increased or decreased in comparison with the initial results (before neutrality analysis) are boldfaced or underlined, respectively. The issues for which neutrality analysis was not able to find any alternative ground truth are greyed out

	

It can be seen that DeepFD is the tool that benefited the most from alternative ground truth selection, as its RC results have improved for 10 out of 22 issues for which alternative GT was available, with the average RC increasing from 0.33 to 0.55. DeepFD is followed by Neuralint, whose RC results improved for 8 issues, which made its average RC to go up to 0.36 from 0.19. On the other hand, for DeepDiagnosis and UMLAUT, the RC values have increased in only 4 cases, with a difference between the old and the new RC of 0.6. It can be seen that the comparative performance observed between the pairs of tools on the original ground truth is generally consistent with the results after neutrality analysis, with the exception of Neuralint and UMLAUT. If before the neutrality analysis their RC and $F_3$ scores were identical, after the performance of Neuralint has become considerably higher. These observations are confirmed by the Wilcoxon signed-rank test with p-value of 0.0008 for the comparison between DeepFD and DeepDiagnosis and p-value of 0.016 for DeepFD vs UMLAUT. The difference between DeepFD and Neuralint is not statistically significant (p-value of 0.063). The complete results are available in the replication package (Humbatova et al. 2023).

Overall, our research highlights the importance of considering the existence of multiple potential fault-inducing changes. Fault localisation results change significantly when we broaden the definition of ground truth to include alternative fault-fixing changes.

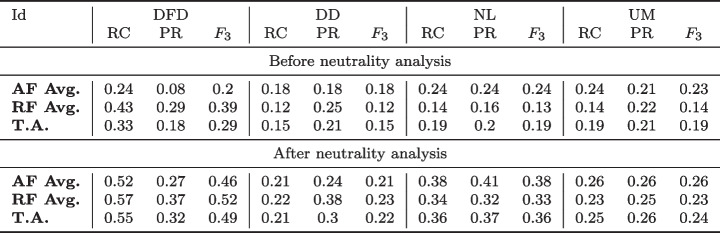



### RQ2 (Stability)

The authors of DeepFD account for the instability of the training process and perform 20 retrainings when collecting input features both during the classifier training stage and during fault identification. This way, the output of the tool is calculated from 20 feature sets for each model under test.

**Table 12:** Overall comparison of Recall (RC), Precision (PR) and $F_3$ measure for each FL tool before/after neutrality analysis. Avg. shows the average within artificial (AF) or real (RF) faults. T.A. shows the total average across faults

	

Neuralint does not require any training and is based on static rules that are stable by design. We performed 20 runs of all other tools to investigate their stability. We found out that outputs are stable across the experiment repetitions for all considered tools.

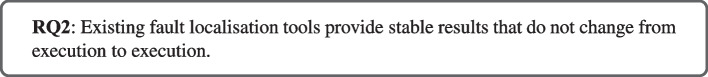



### RQ3 (Efficiency)

In this RQ, we investigate how demanding the evaluated approaches are in terms of execution time. Here we measure only the time required to run an FL tool on a subject, without taking into account the time and effort needed to prepare the subject for the tool application or to post-process the tool output (see Sec. 5.1.1). All tools require some manual work to be done: for DeepFD, a user has to create serialised versions of the training dataset and model configuration according to a specific format; for DeepDiagnosis and UMLAUT, a user has to insert a tool-specific callback to the code and provide it with a list of arguments; for Neuralint, there are a number of manual changes to the source code to make the tool run.

**Table 13:** Execution time (in seconds)

	

**Fig. 2:**
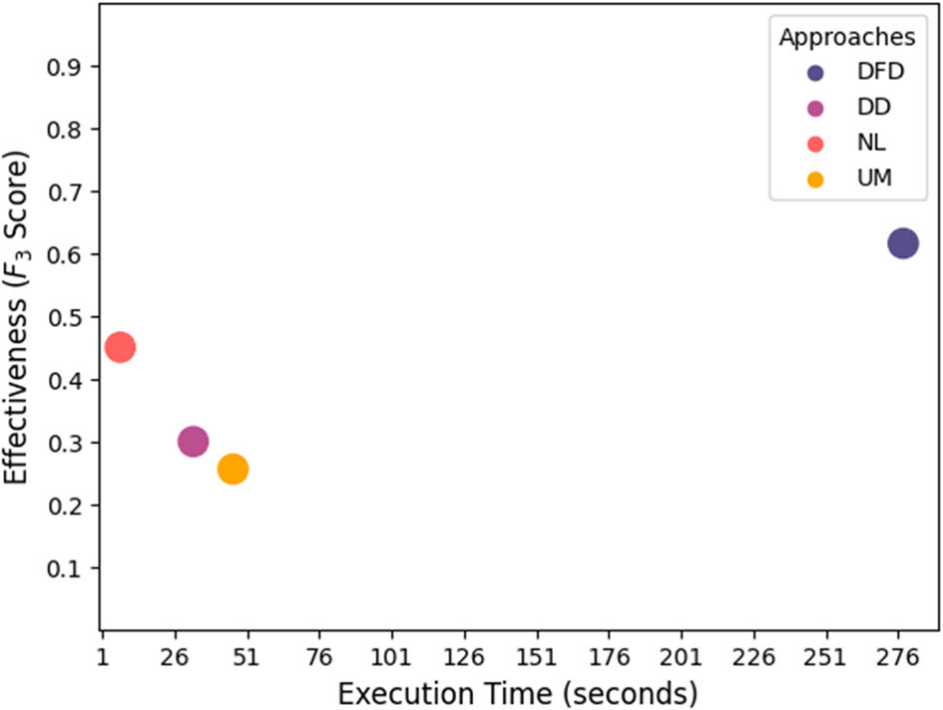
Average execution time and average performance ($F_3$ score) for each tool

Table 13 shows the execution time measured in seconds on a single run of DeepFD and Neuralint, and the average of 20 runs for the remaining tools. Row ‘T.A.’ shows the average time spent by each tool on fault localisation over the whole benchmark. To allow fair comparison, row ‘Avg.’ shows the average execution time over the faults where all tools are applicable. Not surprisingly, DeepFD takes considerably longer to run than the other tools, as it fully trains 20 instances for each issue, while the other tools perform one (DeepDiagnosis, UMLAUT) or no retraining (Neuralint). In addition, DeepDiagnosis often terminates the training when a faulty behaviour is observed, which makes its average execution time the shortest among the tools that require model training. As Neuralint does not require training a model to perform fault localisation, its average execution time is the lowest. It can be noted that for some faults that are very fast to train (e.g. C2, D1, D3, D6, D8), a full training performed by UMLAUT takes less time than the static checks of Neuralint. On average, Neuralint is the fastest to run, followed by DeepDiagnosis and UMLAUT, and finally DeepFD. Despite the differences, the execution time of all tools considered is compatible with real-world use. In Fig. 2, we show the average execution time of each tool combined with the average performance measured using the $F_3$ score. A longer execution time is compensated for in terms of greater effectiveness in the case of DeepFD, while this is not the case for DeepDiagnosis and UMLAUT, which are outperformed by the extremely efficient Neuralint.

We also performed a statistical analysis (Wilcoxon rank sum test) of the execution times. In particular, the differences in the execution times are statistically significant (p-value less than 0.05) in all cases but for the D16 run with NL and UM (p-value $= 0.105$). We did not perform a statistical test of the run time between DFD and NL since both tools were run only once. The corresponding results are also available in the replication package (Humbatova et al. 2023).

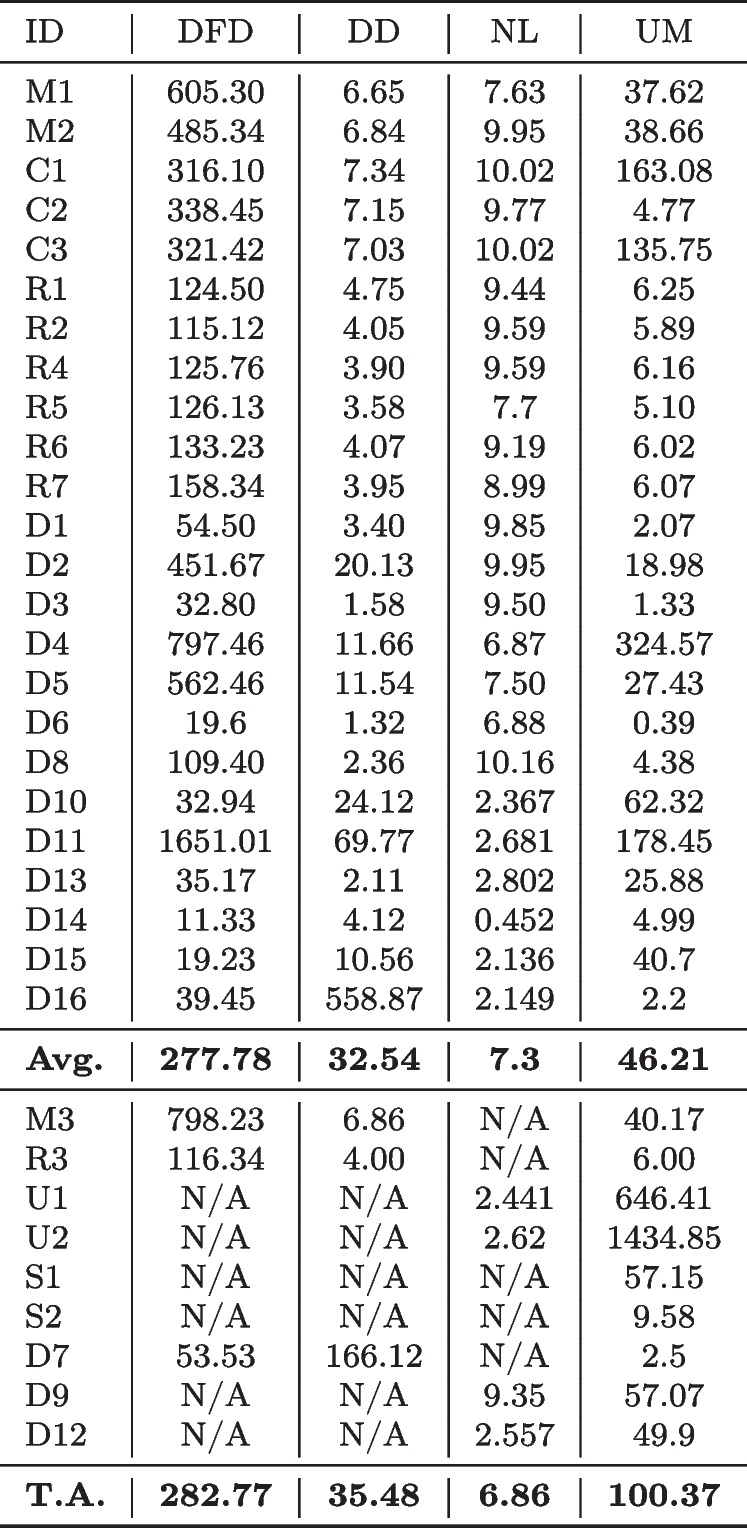



### The Outputs of Fault Localisation Tools

The analysed fault localisation tools output messages in natural language that explain where the fault is present in the neural network. The level of understandability of these messages, as well as the degree of detail they provide about the location of the fault and its possible fixes, is an important indicator of the applicability of these tools in practice. We have analysed each output message produced by each tool and provide an overview of our findings in this subsection.

The output of DeepFD consists of a vector of parameters from the following list [*‘optimizer’, ‘lr’, ‘loss’, ‘epoch’, ‘act’*]. In our experiments, the size of the output vector varied between 1 and 4. While the provided vector clearly indicates which hyperparameters might contain faulty values, it provides no indication on how the values should be changed, e.g., whether the learning rate should be increased or decreased. Moreover, for the activation hyperparameter, the layer number for which the activation function should be changed is not indicated.

The outputs of DeepDiagnosis often refer to problems observed for internal parameters during the training process. Some of these outputs, such as “*Batch 0 layer 6: Numerical Error in delta Weights, terminating training*”, do not provide any guidance on what should be changed in the model architecture, training data or hyperparameters to fix the fault. In contrast, some other outputs such as “*Batch 0 layer 9: Out of Range Problem, terminating training. Change the activation function to softmax*” or “*Batch 0 layer 0: Vanishing Gradient Problem in delta Weights, terminating training. Add/delete layer or change sigmoid activation function*” are more instructive and provide layer numbers along with the required changes.

UMLAUT provides an output that lists critical issues as well as warnings, e.g., “<*Critical: Missing Softmax layer before loss*>*,* <*Warning: Last model layer has nonlinear activation*>” and “<*Critical: Missing Softmax layer before loss*>*,* <*Critical: Missing activation functions*>*,* <*Warning: Last model layer has nonlinear activation*>”. It should be noted that in our experiments UMLAUT reported the critical issue of “*Missing Softmax layer before loss*” for all the analysed faults, including the cases when the softmax layer is already present in the model architecture. Messages indicate the layer number (“before loss” or “last model layer”) in some cases, while in others this information is missing (“<*Critical: Missing activation functions*>”). The latter error is raised also when the activation function is specified not in the layer itself, but in a subsequent specific ‘Activation’ layer. Similarly to DeepDiagnosis, some of the warnings produced by UMLAUT do not contain actionable fix suggestions, for example, “Possible over-fitting” or “Check validation accuracy”.

The output messages of Neuralint report faults either in specific layers (“*Layer 4 ==*>* The initialization of weights should not be constant to break the symmetry between neurons*”) or in the learning process (“*Learner ==*> *The loss should be correctly defined and connected to the layer in accordance with its input conditions (i.e., shape and type)-post_activation*”). The messages provide information on which component is faulty, along with an explanation of why it is faulty.

Overall, except for some cases in DeepDiagnosis, the outputs of FL tools provide clear messages indicating which types of hyperparameters are faulty. However, in cases when the hyperparameter can be applied to different layers of the model, the localisation to the specific layer is not always performed. When it comes to fix suggestions, while DeepFD provides no information in this direction, the remaining tools have some output messages that come with suggested repairs.

## Discussion and Implications

In this work, we perform the evaluation of existing FL approaches on a set of carefully selected issues. Although some of the tools compared their results with those of the existing approaches in the corresponding publications, such comparisons were limited, and used ad-hoc metrics and tool-specific benchmarks. The goal of this study is to provide a fair and standardised comparison of the fault coverage of such tools, their effectiveness, and efficiency. The results are aimed at guiding developers in selecting an appropriate tool for their specific situation, which might be dictated by the resources available or model type and architecture. In this section, we provide some insight for future research on the implementation and evaluation of DL testing tools. In particular, we discuss the importance of a properly collected evaluation benchmark and best practices in collecting reproducible faults. Finally, we convert the results and observations of this study into suggestions for directions of future research.

### Ensuring Quality and Reliability in Benchmarking

Evaluation benchmarks play a crucial role when it comes to the evaluation and comparison of DL testing tools. They act as proxies for real-world scenarios, simulating the kinds of faults and model architectures that practitioners encounter. The quality and characteristics of such benchmarks significantly influence the reliability and generalisability of conclusions drawn from experimental results. Thus, ensuring quality and representativeness of faults enables meaningful comparisons between tools and facilitates the replication of experiments by other researchers. Without well-designed benchmarks, it would be challenging to assess the true capabilities of a testing tool or to identify its strengths and limitations in various contexts. Ideally, a comprehensive benchmark would meet the following characteristics:

- *Representativeness*. Benchmark issues should cover a range of different tasks (e.g., classification and regression), popular frameworks, architectures, complexity, fault types, and domains of application. Such requirements ensure that the evaluated tools are judged on their ability to handle diverse real-world scenarios rather than a narrow subset of problems or widely-used toy models.
- *Realism*. Faults within the benchmark programs should reflect bugs commonly encountered in DL development.
- *Reproducibility*. Faults should be obtained from verifiable sources using a strict methodology that would ensure the benchmark’s correspondence to the real-world issues reported by developers. Faults should be also well-documented, by including both faulty and fixed versions of the program, and all the necessary dependencies to replicate the execution environment where the fault can be exhibited.
- *Independence*. Preferably, the evaluation benchmark should be independent from the evaluated tool, to provide a standardised background for fair comparisons.

Previous work (Jahangirova et al. 2024) revealed that only a small number of issues used in the evaluation of DNN testing techniques (Cao et al. 2022; Wardat et al. 2022; Nikanjam et al. 2021) actually meet these quality and reproducibility criteria. In future work, researchers could build on the benchmark used in this study or create their own fault benchmark while adhering to these criteria. In our work, we proposed neutrality analysis as a way to augment existing benchmarks and expand the evaluation of DNN FL tools. Introducing alternative GTs can make the evaluation more comprehensive and fair. We argue that all future research should consider alternative GTs as part of the evaluation approach.

### Practical Selection of FL Tools

Our findings offer valuable insights for practitioners in selecting the most suitable FL tool for their specific needs. Existing DL fault localisation approaches cover two types of approach to fault detection: dynamic and static. DeepFD and DeepDiagnosis rely exclusively on dynamic analysis, and Neuralint only uses statically available information, while UMLAUT benefits from both approaches.

Another crucial factor in selecting a tool might be the Python and Tensorflow versions of the project under test. In particular, DeepDiagnosis works only with code compatible with Tensorflow 1 and all the remaining tools work with Tensorflow 2. Tensorflow 1 is normally supported by Python versions no higher than 3.7, while Tensorflow 2 is tested and supported by Python 3.8-3.11 (Abadi et al. 2015).

On the other hand, the application domain and available resources might play an important role in choosing testing tools. Practitioners working in a safety-critical domain might prefer tools with higher recall, despite longer execution times, to ensure comprehensive fault coverage. Conversely, in time-sensitive environments or when models take a very long time to train, tools with faster execution times, i.e., relying on static analysis, may be preferable. For instance, while DeepFD achieves high recall, it requires training multiple instances to account for randomness in the training process, which may not always be feasible in resource-constrained environments. However, it is important to note that in the original publication, the authors used 10 retrainings of the model, while in our experiments we performed 20 retrainings.

In addition, our study identified some setup challenges associated with specific tools, such as the need for manual source code modifications. Usage of DeepDiagnosis implies editing the framework’s source by adding its proprietary callback and then initialising this callback before the model training while providing a number of model parameters such as the number of layers or learning rate, before passing the callback to the ‘fit’ function. Similarly, UMLAUT requires its callback to be initialised and passed to the model for training. Additionally, this tool would not run if the validation data is not presented to the ‘fit’ call and if ‘accuracy’ is not in the list of the calculated metrics. DeepFD calls for a more sophisticated preprocessing like serialising the configurations of the model (loss function, optimiser, learning rate, number of epochs and batch size) and the whole training and validation datasets. It also requires the faulty model to be trained and saved in the ‘h5’ format. Neuralint parses the source code of the deep learning model and builds a graph that is then used to verify the pre-determined rules. Therefore, its usage is hindered by the limitations of the parser. Very often to make Neuralint applicable to the source code, the source code needs to be simplified or to follow some specific structure. For example, if the layer is indicated as a separate layer (which is something the Keras framework allows), the source code would not be parsed. When running our experiments, we discovered some of these limitations. Practitioners should weigh these factors against tool performance metrics to make informed decisions about adoption.

### State of the Art and Future Directions of DNN Fault Localisation

Our findings reveal that none of the considered tools can successfully detect and localise DL faults, which indicates that FL for DNNs is still an unsolved problem. In fact, existing tools cover only 27% to 60% of the fault types encountered in our benchmark, which is only a fraction of the real DL fault types reported in real DL fault taxonomies (Humbatova et al. 2020). Researchers might consider using state-of-the-art fault classifications and tool coverage reported in this study as a guidance when designing future FL tools.

The majority of existing FL tools focus on models developed exclusively using the Keras framework, while Tensorflow and Pytorch, other extremely popular DL frameworks (Hale 2018), have not been considered. The only exception is Neuralint that is also applicable to Tensorflow. Moreover, current approaches that use a dynamic approach are only applicable to simpler ’Sequential’ Keras models (Abadi et al. 2015), failing on more flexible ’Functional’ architectures (Abadi et al. 2015). Additionally, none of the approaches can handle models using custom (non-native to Keras framework) loss functions and multiple model inputs. These observations indicate that as the field of DL evolves, more diverse and sophisticated tools are required to assist practitioners in the development process.

Another important factor for future research is the maintainability of the tools. Programming languages such as Python, which is widely used for DL programming (DeepLearning.Al 2024), and popular DL frameworks (Hale 2018), periodically release newer versions and deprecate or abolish old functionality and versions. This makes FL tools inapplicable to software developed with a more recent codebase and might hinder their practical and academic usage.

Lastly, it is important to consider the scalability of the tools for practical use. Many modern and industrial models have billions of parameters and a large amount of training data. When implementing novel FL tools, scalability to such size should be taken into consideration to allow smooth and affordable integration of a tool in the tested system environment.

## Threats to Validity

### Construct

A potential threat to construct validity in our study lies in the metrics used to evaluate the effectiveness of fault localisation tools. To address this, we employed a straightforward count of matches between the fault localisation results and the ground truth, supplemented by standard metrics from information retrieval, including Recall (RC), Precision (PR), and $F_{\beta }$, to ensure a well-rounded assessment.

Another potential threat to construct validity is the existence of multiple plausible ground truth (GT) repairs for faulty deep learning models. When a fault is identified, there may be several valid ways to repair a model, and these alternative fixes can vary significantly in their structure, behaviour, or performance. This introduces ambiguity in evaluating FL tools, as they may propose repairs that deviate from the selected GT but are equally valid or even superior. To mitigate this, we adopted an approach to identify a set of alternative patches that can serve as a set of possible ground truth fixes. We then report the performance of the FL tools across this set.

### Internal

One threat to internal validity of the study lies in the selection of evaluated FL tools. To the best of our knowledge, we considered all state-of-the-art techniques and adopted their publicly available implementations. Another potential threat is the randomness inherent to the underlying DL systems and possibly present in the FL tools. To account for this threat, we have evaluated the stability of the FL tools that are affected by randomness as part of RQ2 by running the tools 20 times. Finally, a potential threat to internal validity is the fault injection methodology, as inconsistencies or biases in fault injection could unfairly advantage or disadvantage specific tools. To mitigate this, we used an independent fault injection tool, DeepCrime (Humbatova et al. 2020), ensuring that the fault injection process is systematic and unbiased.

### External

The primary threat to external validity in our study is the representativeness of the fault benchmarks used. To mitigate this, we incorporated both real faults and artificial faults. For the real faults, we relied on the dataset curated by Jahangirova et al. (2024), which aggregates faults from five different benchmarks. These faults were carefully filtered to ensure they meet realism criteria and are reproducible. To generate artificial faults, we applied nine different mutation operators to six distinct DL models, each performing a task in a different domain. Despite these efforts, replicating our study with additional subjects and datasets would further validate and strengthen our findings.

## Related Work

While to the best of our knowledge ours is the first empirical study that performs a third party assessment of existing DL fault localization tools, there is a previous empirical work (Kim et al. 2023) aimed at comparing different DL repair approaches. In the following, we first discuss such empirical work, followed by a summary presentation of existing repair approaches: although they do not address the DL fault localisation problem, they are relevant to such task.

The DNN model architecture repair problem, as defined in the recent study by Kim et al. (2023), lies in improving the performance of a faulty deep neural network (DNN) model by finding an alternative configuration of its architecture and hyperparemeters. The new configuration should lead to a statistically significant enhancement in model performance, such as accuracy or mean squared error, when measured on a test dataset. In particular, the authors consider a number of categories and subcategories from a DL fault taxonomy (Humbatova et al. 2020), covering the following issues: faults affecting the structure and properties, faults affecting the DNN layer properties and activation functions, faults due to missing/redundant/wrong layers, and faults associated with the choice of optimiser, loss function and hyperparameters (e.g., learning rate, number of epochs) as model architecture faults. Examples of such faults include the selection of an inappropriate loss function for the task at hand or training a model for an insufficient number of epochs.

Existing advances in Hyperparameter Optimisation (HPO) can be considered as a way to address the problem of repair as they can be applied to search optimal configurations for different aspects of model architecture such as activation functions, number of neurons and layers, hyperparameters affecting the training process, etc. At the moment, there is no automated *source-level* repair tool that improves performance of a model by means of patching and modifying the sources of the model’s architecture. However, there exists a tool called AutoTrainer (Zhang et al. 2021) which is designed to detect and repair training problems such as dying ReLU or exploding gradients, by continuing the training with patched architecture or hyperparameters.

Kim et al. (2023) compared AutoTrainer with HEBO (Cowen-Rivers et al. 2022) and BOHB (Falkner et al. 2018), state-of-the-art HPO techniques based on Bayesian Optimisation (BO), while using random search as a baseline. The comparison was performed on a carefully compiled set of artificial and real-world faulty models. Their results demonstrate that the evaluated techniques can potentially improve the performance of models affected by architecture faults. However, their findings indicate that there is still considerable room for improvement as random baseline performs quite well when compared with other techniques.

On the other hand, there exist a number of post-training *model-level* repair approaches that focus on modifying the weights of an already trained model in order to eliminate observed misbehaviours. Arachne (Sohn et al. 2022) and Care (Sun et al. 2022) both focus on identifying the neurons that contribute the most to the detected misbehaviours on certain test inputs, and calibrate the weights associated with these neurons, while trying not to corrupt correct predictions. GenMuNN (Wu et al. 2022), however, directly locates the weights that play the biggest role in predictions, and uses a genetic algorithm to evolve the model by applying slight mutations to such weights. I-Repair (Henriksen et al. 2022) also locates and changes the weights that take part in forming a misbehaving output for a certain group of inputs, while maintaining the same behaviour on correctly classified inputs. PRDNN (Sotoudeh and Thakur 2021) similarly aims at producing the smallest achievable single-layer repair. NNrepair (Usman et al. 2021) uses constraint solving to produce slight modifications to suspicious weights revealed by fault localisation. Apricot (Zhang and Chan 2019) adjusts the weights of a misbehaving model using the guidance from the weights of a complementary correctly-performing model trained on reduced dataset that contains the problematic inputs.

While hyperparameter optimisation tools provide source level information about the performed fixes, which means they also offer some fault localisation capability, post-training repair tools are completely opaque and their fixes have no interpretation in terms of architectural model elements affected by a fault. In our empirical study, we restricted the selection of tools to those that explicitly address the DL fault localisation problem.

## Conclusion

We evaluated four state-of-the-art techniques in DL fault localisation on a meticulously tailored set of real and artificial faulty models to assess the advances in the area. Our findings show that all of the evaluated approaches are able to locate a certain percentage of faults. However, all are quite far from the best possible results when considering the available ground truth. DeepFD exhibited the highest effectiveness, followed by Neuralint and UMLAUT. DeepDiagnosis exhibited relatively poor performance. On the positive side, all proposed techniques are stable across multiple runs and do not require excessive execution time. However, our experimentation suggests that when re-computing the results after including multiple alternative to ground truth patches (obtained by neutrality analysis), the FL accuracy of tools increases in all cases, sometimes quite substantially.

According to our findings, future work in the area of DL fault localisation should focus on improving the fault identification capabilities of the proposed techniques and broadening the variety of considered fault types. Moreover, any empirical evaluation of DL fault localisation tools should include some form of neutrality analysis, to expand the available ground truth to other possible, equivalent fixes.

## Data Availability

The experimental data, code, and evaluation results supporting the findings of this study are available on the Zenodo platform (Humbatova et al. 2023) with the following identifier: 10.5281/zenodo.10387015.

## Footnotes

1 A model is considered *neutral* relative to another model if it has equivalent or higher fitness than the other’s, by comparing the mean accuracy of ten trained instances of the model and the other model.

2 For the computation of statistical significance, we employ a Generalized Linear Model (GLM) with a significance level 0.05 and Cohen’s *d* to measure the effect size, for which we adopt a threshold at 0.5.
